# Children’s experiences of living with their mental ill-health - a scoping review

**DOI:** 10.1080/17482631.2025.2501682

**Published:** 2025-05-07

**Authors:** Eva-Karin Gotting, Laura Darcy, Åsa Israelsson-Skogsberg, Annelie J. Sundler, Ewa Carlsson Lalloo

**Affiliations:** Faculty of Caring Science, Work Life and Social Welfare, University of Borås, Borås, Sweden

**Keywords:** Children, mental ill-health, qualitative research, scoping review, Well-being, lived experiences

## Abstract

**Purpose:**

This study aims to identify and summarize existing qualitative empirical research on children’s experiences of living with their mental ill-health.

**Methods:**

A scoping review with a systematic search of the databases PubMed, CINAHL, and PsychINFO was conducted. The search generated 9,864 studies, which were screened by title, abstract, and full text.

**Results:**

Forty articles were included comprising 826 children aged 8–19 years. The key findings were described in four themes: *Identifying oneself with mental ill-health, Managing suffering and daily challenges, Seeking supportive and caring relationships* and *Navigating a complex school environment*. Being identified with mental ill-health brought challenges for children’s everyday struggles. Their own coping strategies as well as supportive relationships with friends and adults were important. However, the balance between support and stress was complex.

**Conclusions:**

Children have a desire to manage and comprehend their complex everyday lives of living with mental ill-health and wish for supported yet independent decision-making. Attitudes of friends, adults and professionals are important in providing understanding and non-judgemental support, valuable for children’s well-being. Open conversations about mental health and providing accessible, child-centred interventions based on the needs expressed by children are necessary. This study contributes to the literature by emphasizing the central role of children’s voices in matters of mental ill-health.

## Introduction

Childhood is a formative period shaped by environmental interactions, including family dynamics, school settings and peer relationships (Bronfenbrenner, 1979; Housman, 2017). The United Nations Convention on the Rights of the Child (UNCRC) (1989) defines a child as anyone under eighteen and emphasizes the importance of protecting children’s well-being and ensuring their voices are heard (Unicef, 2022). Globally, mental ill-health among children and adolescents is a growing concern (Potrebny et al., 2017). Nearly half of all mental ill-health conditions arise in childhood and adolescence (Solmi et al., 2022) and are well-known to have negative consequences for children’s education, quality of life, well-being and social functioning (Patel et al., 2007; Qualter et al., 2013). While diagnoses such as anxiety and depression are increasingly recognized, children do not always receive the help needed (O’Brien et al., 2016; Reardon et al., 2018).

For children with mental ill-health, there may be barriers to accessing specialist services and treatment (O’Brien et al., 2016). Families and children report experiencing problems with these services related to lack of access to care, inadequate communication, services not addressing the children’s needs and putting responsibility on parents (Darcy, Råberus et al., 2024). These kind of barriers to receiving care contribute to a sense of helplessness among parents, as they struggle to find appropriate support for their children’s mental ill-health. Parents seek treatment approaches that are personalized to their child’s individual needs and circumstances (Salamone-Violi et al., 2017). This aligns with the principle of child-centred care where it is crucial to incorporate both a child perspective—informed by adults’ intensions regarding children’s welfare—and the child’s perspective, which values children’s own experiences and opinions (Söderbäck et al., 2011).

Understanding how children themselves perceive and navigate mental ill-health remains underexplored. To contribute to future positive development, we need to understand individual children’s experiences and problems related to mental ill-health to guide early interventions and comprehensive care. Understanding children’s lived experiences of mental ill-health are important for informing clinical practice and mental health literacy interventions (Georgakakou-Koutsonikou & Williams, 2017). A deeper understanding of children’s perspectives and experiences is also necessary to address barriers to help-seeking.

A recent review highlights the complex nature of mental health as described by children and youths (Beckman et al., 2023). The descriptions of uncertainty and conflicting perceptions in their understanding of mental health highlight a knowledge gap regarding a shared language concerning mental health, essential to differentiate between everyday challenges and issues that require treatment. Children often struggle to differentiate between the severity of mental health problems and everyday stressors or challenges. This indicates a pressing need to better understand how mental ill-health manifests in children, specifically through the lived experiences of children. By addressing these gaps, a more comprehensive understanding of children’s mental health can be developed to improve care practices.

### Aim

To identify and summarize existing qualitative empirical research on children’s experiences of living with their mental ill-health.

## Materials and methods

This scoping review was conducted in accordance with the Joanna Briggs Institute (2020) methodology for scoping reviews and followed the Preferred Reporting Items for systematic Reviews and Meta-Analyses extension for Scoping Reviews (PRISMA-ScR) (Tricco et al., 2018). A scoping review was chosen to map existing literature, identify key concepts and research gaps, making it more suitable than a systematic review for this broad topic (Joanna Briggs Institute, 2020). The completed PRISMA-ScR checklist for this study is provided as Supplementary file 1. The protocol for this review was preregistered in Open Science Framework (OSF), ID:https://doi.org/10.17605/OSF.IO/XGZVB. The scoping review process was structured according to Arksey and O’Malley’s (2005) five stages: identifying the research question, identifying relevant studies, study selection, charting the data and collating, summarizing and reporting the results.

### Stage 1: identifying the research question

The concepts of Population, Concept and Context (PCC), see Table I, were applied to identify the aim and facilitate the identification of existing knowledge within the research field as well as provide an overview, in accordance with the scoping review methodology outlined by Arksey and O’Malley (2005), and later refined by Levac et al. (2010) and Peters et al. (2015). The population of children were defined as all children and adolescents 0–19 years of age, including teenagers but excluding young adults.

**Table I.** Population, concept and context (PCC).

Component	Content	Definition or inclusion criteria	Aim
Population (P)	Children	Children and adolescents (to include teenagers), 0 - ≥ 19 years of age	To identify and assess existing qualitative empirical research on children’s experiences of living with their mental ill-health
Concept (C)	Children’s experiences	Own experiences from living with mental ill-health	
Context (C)	Mental ill-health	From general population and healthcare settings	

### Stage 2: identifying relevant studies

A query-string was established based on an iterative process, conducted in collaboration with a research librarian. The query-string included terms to narrow the search to experiences of children living with mental ill-health and was tweaked and re-evaluated throughout a pilot search. Keywords were used for each PCC concept during the search process: For Population (P) terms such as “children” and “adolescents” were applied. For Concept (C), keywords included “experiences”, “perspectives” and “attitudes”. For Context (C), terms like “mental ill-health” and “mental health” were used. Boolean operators such as AND and OR were applied to refine the search. The final query-string with MeSH-terms, was tailored for each database-Cinahl, PubMed and PsychInfo, see Supplementary file 2.

### Stage 3: study selection

Inclusion criteria were scientifically peer-reviewed, qualitative studies with children aged 0–19 years living with self-reported or diagnosed mental ill-health. Language and time restrictions were limited to English and studies performed between 2000 and 2024. Exclusion criteria were studies that involved adults reporting on children’s experiences with mental ill-health and studies with quantitative or mixed methods. Studies exploring general mental ill-health among children during the COVID −19 pandemic, or studies that evaluated interventions/experiences of certain treatment or medications, were excluded. Studies that reported participants who were in the risk-zone for mental ill-health but had not yet developed mental ill-health were also excluded.

The literature search generated 12 794 studies. Duplicates were identified and removed using Rayyan, a web-based software systematic review tool for screening management (Ouzzani et al., 2016). After the removal of duplications 9864 studies were screened first for title and then for abstract. To validate the screening procedure, three authors independently screened a total of 50 studies each by title to compare consistency. The screening process was divided as follows: the first author screened all titles, while ECL and LD jointly screened half of them independently. Disagreement was solved by AS and ÅI-S. Following the title screening, the abstracts of 2819 studies were screened. To ensure agreement in the inclusion and exclusion of relevant studies, the same three authors independently screened another set of 50 abstracts. As before, the first author screened all abstracts, while ECL and LD jointly screened half of them independently. Any disagreements were discussed among all authors to reach reliability, before inclusion or exclusion.

**Figure 1.**
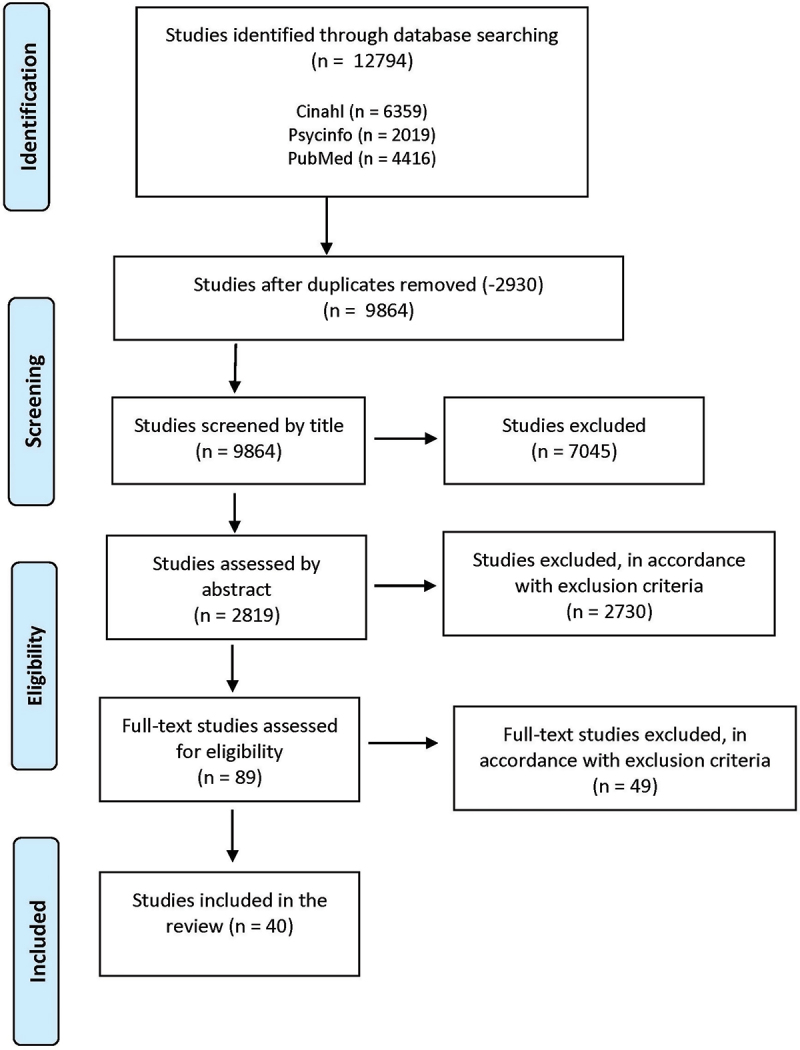
Flow chart of the literature search and screening according to PRISMA (Tricco et al., 2018).

After the abstract screening, the full-texts of 89 studies were assessed. The first author, ECL and LD, independently screened all of these and thereafter a total of 40 studies were included in the review. A flow chart of the literature search and screening of studies is shown in Figure 1. All included studies were assessed for quality and met criteria using the Mixed Methods Appraisal Tool (MMAT) (Hong et al., 2018).

### Stage 4 and 5: charting the data, and collating and summarising the results

Data were extracted from the included studies primarily by the first author using a predefined extraction form, see Table II. To ensure its applicability, the form was pilot tested on five studies by the first author and ECL independently. Following this test, the first author proceeded with data extraction. The extracted data included specific details about the authors, year, country, aim, methods and participants. Key findings on children’s experiences of mental ill-health, relevant to the research question, were also extracted. Given the substantial volume of qualitative data, thematic analysis was employed, following the six-phase approach described by Braun and Clarke (2022). Meaning-bearing units relevant to the study’s aim were extracted from five studies by two authors independently and then the first author continued this process with remaining articles. In the next phase all meaning bearing units were clustered. These findings were read several times and codes were developed through an iterative and inductive process, where meaningful units related to the research question were identified. The analysis was conducted by the first author. The entire author group compared and discussed codes and themes to achieve consensus, a process carried out during supervision meetings. This approach focused on identifying patterns, in line with guidance from Aveyard (2023), maintained focus on the scoping review methodology and avoid deeper interpretative analysis. The results were then organized into themes and subthemes with descriptive summaries, as recommended by Joanna Briggs Institute (2024).

**Table II.** Characteristics and summary of included studies.

Authors/Year/Country	Aim	Methods	Participants
Amrtavarshini et al. (2024)/India *Evolution of anhedonia in adolescent depression: An interpretative phenomenological analysis study*	Get an in-depth person-centred perspective on the experience of anhedonia in adolescents with depression	Semi-structured interviews. Interpretative Phenomenological Analysis (IPA)	10 girls aged 15–18 with moderate to severe depression, were on regular antidepressant medications, and had individual psychotherapy.
Arbour et al. (2023)/Canada *Exploring the Recovery Phenomenon from Adolescents’ Perspective: A Qualitative Study*	Understand the recovery phenomenon from the perspectives of adolescents.	Interviews with phenomenological approach	9 children, 7 girls and 2 boys, between 14 and 18 years with a variety of diagnoses including but not limited to eating disorders, psychosis, mood disorders, anxiety disorders, and/or depression.
Bird et al. (2022)/UK *The journey of adolescent paranoia: A qualitative study with patients attending child and adolescent mental health services*	Explore the development, experience, and impact of paranoia in adolescent patients.	A qualitative interview design with interpretative phenomenological analysis	12 children, 9 girls and 3 boys, 11–17 years—median age 15,5 years mean age 14,7, with paranoia.
Corser et al. (2023)*/UK* *A whirlwind of everything’: The lived experience of adolescents with co-occurring chronic pain and mental health symptoms*	Examine the lived experience of adolescents with co-occurring chronic pain and mental health symptoms.	Semi-structured personal communication, interpretative phenomenological analysis.	7 girls, 15–18 years, self- reporting diagnoses of both pain and mental health issues for a duration of 3 months or longer.
Dardas et al. (2019)/Jordania *Depression in Arab Adolescents*	Capture adolescents’ experience of depression, identify perceived contributing factors and assess their attitudes towards depression interventions	An exploratory, qualitative design was used to collect data from 12 focus groups.	92 children, 56 girls and 36 boys, aged 14 to 17, with reported experiencing mild to moderate symptoms of depression.
DeFosset et al. (2017)/USA *Youth Descriptions of Mental Health Needs and Experiences with School- based Services: Identifying Ways to Meet the Needs of Underserved Adolescents*	Explore how youth express mental health symptoms, and their trajectories through, and perceptions of, school- based mental health services.	A qualitative descriptive approach was used to analyse data from in-depth interviews.	18 children, 13 girls and 5 boys, aged 12–18, who reported experiencing mental health problems affecting their daily functioning, including but not limited to, their ability to attend school.
Elsina and Martinsone (2020)/Latvia *Interpersonal Relationship Aspects as Perceived Risk and Social Support Factors in a Clinical Sample of Adolescents With Depression*	Explore how adolescents with diagnosed depression describe their social relationships with peers, parents and teachers.	Individual semistructured interviews, analysed using thematic analysis.	28 children, 20 girls and 8 boys, aged 13–17, with a diagnosis of depression.
Farmer (2002)/USA *The experience of major depression; adolescents’ perspectives.*	Describe the experience of major depression from the adolescent’s perspective.	In-depth interviews were conducted with a phenomenological approach. Data analysis with an adaption of Colaizzi’s method.	5 children, 3 girls and 2 boys, aged 13–17 with depression.
Gampetro et al. (2012)/USA *Life Concerns and Perceptions Of Care in Adolescents with Mental Health Care Needs: A Qualitative Study In a School-Based Health Clinic*	Explore the perceptions of mental health needs of teens.	A single, face-to-face, semi-structured interview was used to examine students’ concerns and attitudes towards their health care needs and services.	18 children, 10 girls and 8 boys, between 12 to 18 years of age with diagnosed behavioural or mental health issues.
Halldorsson et al. (2023)/UK *In the moment social experiences and perceptions of children with social anxiety disorder: A qualitative study*	Examining the social experiences of children with Social Anxiety Disorder (SAD).	Reflexive thematic analysis to analyse the transcripts of interviews with children with SAD who had been interviewed about their “in the moment” social experiences during a social stress induction task.	12 children, 8 girls and 4 boys, aged 8–12 years with SAD.
Higson-Sweeney et al. (2024)/UK *“I’m always going to be tired”: a qualitative exploration of adolescents’ experiences of fatigue in depression*	Explore adolescents’ experiences and understandings of fatigue in depression.	Semi-structured interviews. A Young Person’s Advisory Group, comprised of young people with current or previous experience of depression, was consulted at every stage of this study, from design to dissemination.	19 children, 11 girls, 5 boys, 3 non-binary, 1 transgender, aged 14–18 years old with elevated symptoms of depression.
Klauber et al. (2024)*/Denmark*. *I Didn’t Want the Psychotic Thing to Get Out to Anyone at All: Adolescents with Early Onset Psychosis Managing Stigma*	Explores adolescents’ experiences of psychosis stigma.	Semi-structured interviews, phenomenological analysis.	34 children, 23 girls and 11 boys, aged 14–19 years, with a diagnosis of psychosis.
Kline et al. (2023)*/USA* *Adolescent Experiences With Social Media and Suicidality*	Investigate adolescents’ experiences with social media and its role in the presentation for suicidal behaviours at the point of a mental health emergency.	Qualitative interviews. Using grounded theory, data collection proceeded along with cultivation of themes until thematic saturation was achieved.	17 children, 10 girls and 7 boys where 82% identified as cisgender, aged 13–17, presenting to the emergency department for suicidal ideation and suicide attempts.
Kranke et al. (2011)/USA *A Qualitative Investigation of Self-Stigma Among Adolescents Taking Psychiatric Medication*	Elucidate youths’ responses to mental illness labels and how psychiatric services affect self-image and self-efficacy	Semistructured interviews.	27 children, 9 girls and 18 boys, aged 12–17 with a mental illness diagnosis.
Kranke et al. (2012)/USA *What Do African American Youth With a Mental Illness Think About Help-Seeking and Psychiatric Medication?: Origins of Stigmatizing Attitudes*	Explore the origin of stigmatizing attitudes among African American adolescents with psychiatric disorders.	A semi-structured instrument, the Teen Subjective Experience of Medication Interview (TeenSEMI), was used to obtain narrative data.	17 children, 11 girls and 6 boys, aged 12–17 with psychiatric disorders.
Latakienė and Skruibis (2015)/Lithuania *Attempted suicide: qualitative study of adolescent females’ lived experience*	Describe the lived experience of attempted suicide among young females.	Semi-structured interviews. Interpretative phenomenological analysis.	3 girls aged 13 to 17, recruited from a psychiatric hospital; diagnoses were not taken into account because of phenomenological grounds of the research.
Li et al. (2022)/USA *Mapping the journey from epistemic mistrust in depressed adolescents receiving psychotherapy*	Explore the phenomenon of epistemic trust and mistrust in depressed adolescents receiving psychotherapy.	45 semistructured interviews at three time points were conducted.	15 children, 12 girls and 3 boys. Aged 11–18 who entered treatment with indications of epistemic mistrust or hypervigilance.
McQueen and Henwood (2002)/UK *Young men in “crisis”: attending to the language of teenage boys’ distress*	Report research on how young men in contemporary Britain talk about their experiences of emotional distress.	Interviews using narrative, thematic and discourse analysis.	2 boys, aged 14–17 years, attended an adolescent unit with different patterns of emotional distress.
Midgley et al. (2016)*/UK* *Just like talking to someone about like shit in your life and stuff, and they help you”: Hopes and expectations for therapy among depressed adolescents*	Explore hopes and expectations for therapy among a clinical population of depressed adolescents.	Semi-structured interviews, analysed qualitatively using framework analysis	77 children, 55 girls and 22 boys aged 11–17, with moderate to severe depression.
Moses (2010)/USA *Being treated differently: Stigma experiences with family, peers, and school staff among adolescents with mental health disorder*	Examine adolescents’ perceptions of being treated “differently” because of mental health problems by family members, peers, and school staff.	Qualitative analysis of narratives from mixed method interviews.	56 children, 21 girls and 35 boys, aged 12–18, diagnosed with one or more mental disorders.
Mulfinger et al. (2019)/Germany *Secrecy versus disclosure of mental illness among adolescents: I. The perspective of adolescents with mental illness*	Explore personal views of adolescents with Mental Illness (MI) on secrecy and disclosure of their MI.	Six focus groups were recorded, transcribed, and analysed by qualitative content analysis.	39 children, 26 girls and 13 boys, aged 13–18, with MI recruited from Child and Adolescent Psychiatry.
Oliver et al. (2015)*/UK* *All these negative thoughts come flooding in’: how young people with depression describe their experience of rumination*	Examine how young people with depression experience rumination.	Interviews analysed using interpretative phenomenological analysis (IPA).	7 children, 5 girls and 2 boys, aged 16–18 with depression.
*Platell et al. (2023)/Australia* *How parents can help or hinder access to mental health services for young people*	Explore adolescent experiences of accessing and utilizing mental health service in Perth, Western Australia with focus on the adolescent identified influence of parents in accessing and using mental health services.	Qualitative semi-structured face-to-face interviews, analysed thematically.	22 children, 16 girls and 4 boys and two identified as other, 12 children identified themselves as LGBTIQ, aged 14–18 (2 were 15 year, one14 year) Recruited from government and non-government community mental health services and youth services.
Reangsing et al. (2024)*/Thailand* *The experience of Thai adolescents with depression: A qualitative study*	Investigate the experiences of Thai-adolescents suffering from depression.	Semistructured interviews, analysed using interpretative phenomenological analysis.	14 children, 10 girls and 4 boys, aged 15–18 with depression recruited by the school health centre at a secondary school.
Shalanski and Ewashen (2019)*/Canada* *An interpretive phenomenological study of recovering from mental illness: Teenage girls’ portrayals of resilience*	Explore the understandings of resilience from the perspective of teenage girls recovering from mental illness	Interviews with interpretive phenomenology.	5 girls, aged 15–16 recruited from an inpatient psychiatric unit.
Shaw et al. (2009)*/UK* *Depression in Female Adolescents: An IPA Analysis*	Gain an understanding of female adolescents’ own experiences of depression.	Open-ended interviews, explored using Interpretative Phenomenological Analysis	6 girls, aged 14 to 17 years, recruited from a local multi-disciplinary Community Child and Adolescent Mental Health Team (CAMHS).
É. Simões et al. (2021)*/Brazil* *Reasons assigned to suicide attempts: adolescents’ perceptions*	Identify the reasons for attempting suicide from the perspective of adolescents.	Qualitative study conducted with Semi-structured interviews were held in July 2020 using WhatsApp. Data were analysed according to Minayo’s Content Thematic Analysis	10 children, 9 girls and 1 boy, aged between 12–17 who attempted suicide and were attending a Centro de Atenção Psicossocial Infanto-Juvenil.
R. M. P. Simões et al. (2020)*/Portugal* *Characterization of adopted suicidal behaviour and its main influencing factors: A qualitative study with adolescents*	Comprehend better the meanings associated with suicide.	Structured interview with content analysis technique.	33 children, 24 girls and 9 boys, aged 13–18, with suicidal behaviour, hospitalized in a child psychiatry unit.
Singleton et al. (2016)*/UK* *Online social networking and psychological experiences: The perceptions of young people with mental health difficulties*	Explore the interaction between online social networking experiences and wellbeing in young people accessing mental health services.	Data from semi-structured interviews, analysed using Grounded Theory methodology. Youths were co-researchers.	12 children, 9 girls and 3 boys, aged 13–18 recruited from community child and adolescent mental health services.
Stänicke (2021)*/Norway* *The punished self, the Unknown self, and the Harmed self—Toward a more nuanced understanding of Self-harm among adolescent girls*	Explore the lived experience of self-harm as it is related to everyday life and challenges among adolescents.	Interviews analysed by Interpretative Phenomenological Analysis.	19 children, aged 13–18 years whose initial treatment contact at an outpatient clinic offering treatment for children and adolescents.
Tang et al. (2023)*/UK* *Links between mental health problems and future thinking from the perspective of adolescents with experience of depression and anxiety: a qualitative study*	Understand how young people perceive and interpret the impact of mental health conditions on their thinking about the future.	Semi‑structured interviews were transcribed verbatim and subjected to thematic content analysis.	19 children, 16 girls and 3 boys, aged 16–19 with clinical or subclinical depression and/or anxiety.
Vallani et al. (2023)/Canada *The journey from concealment to disclosure of an obsessive-compulsive disorder diagnosis in the high school setting: A qualitative study exploring youth perspectives*	Explore adolescent perspectives on the disclosure process in schools	Semi-structured interviews were conducted and analysed inductively through Interpretive Description.	12 children, 5 girls and 6 boys, 1 non-binary, aged 13–17 with a clinician-confirmed DSM IV/5 OCD diagnosis.
Viduani et al. (2022)/Brazil *The experience of receiving a diagnosis of depression in adolescence: A pilot qualitative study in Brazil*	Explore adolescents’ initial reactions after receiving a clinical diagnosis of Major Depressive Disorder in the context of a neurobiological study of depression in Brazil.	Interviews designed to explore the subjective experience of receiving the diagnosis and the impacts of depression on adolescents’ lives. Framework Analysis was used to analyse the accounts.	8 children, 2 girls and 6 boys, aged 14–16 who were in a current depressive episode.
Wang et al. (2023)/China *Adolescents’ attitudes toward non-suicidal self-injury (NSSI) and their perspectives of barriers to seeking professional treatment for NSSI*	Investigate the attitudes of adolescents towards NSSI, and to explore their perspectives on barriers to seeking professional treatment for NSSI	A qualitative phenomenological design was used to conduct in-depth interviews	17 children, 12 girls and 5 boys aged 12–18 (average age 16,2) with NSSI in a psychiatric hospital in Beijing, China.
Watson et al. (2020)/UK. *Understanding anhedonia: a qualitative study exploring loss of interest and pleasure in adolescent depression*	Examine how young people experience anhedonia in the context of depression.	Semi-structured interviews, thematic analysis.	34 children, 17 girls and 17 boys, aged 13–18, recruited both from community and clinic, all with MIH. Boys were extra recruited from community through one-sex school, because of lack in earlier research.
Weitkamp et al. (2016)/Germany *The Experience of Depression: A Qualitative Study of Adolescents With Depression Entering Psychotherapy*	Explore the experience of depression and the journey into therapy of young people (YP) diagnosed with depression.	Semi-structured interviews analysed using Interpretative Phenomenological Analysis	6 children, 5 girls and 1boy, aged 15–19, mild to moderate depressive episodes.
Wisdom and Green (2004)/USA *“Being in a Funk”: Teens’ Efforts to Understand Their Depressive Experiences*	Initiate discussion about teens’ experiences with depression and design a theoretical framework for further research on the topic.	Modified grounded theory, in-depth individual or focus group interviews.	22 children, 8 girls and 7 boys, aged 14 to 19 years diagnosed with depression, recruited from a non-profit, group model, health maintenance organization.
Woodgate (2006)/Canada *Living in the shadow of fear: adolescents lived experience of depression*	Gain an understanding of what it was like to be an adolescent living with depression.	Interviews and field notes with hermeneutic phenomenology.	14 children, 11 girls and 3 boys, aged 13,5–18 diagnosed with depression were recruited from two outpatient adolescent treatment centres.
Wu et al. (2023)/China *What makes Chinese adolescents “trapped” in severe mental illness? An interactionist perspective on self and identity*	Explore the self and identity perspectives among Chinese adolescents with severe mental illness (SMI), with a focus on their illness experience and subjective meaning of a formal diagnosis.	Interview data, analysed with grounded theory approach.	31 children, 16 girls and 15 boys, aged 13–19,
Zhu et al. (2024)/China *Experiences and Cognitive Characteristics of Non-Suicidal Self-Injury in Adolescents With Depression*	To elucidate the underlying motivations, experiential dimensions, and cognitive perceptions in adolescents with depression and non-suicidal self-injury (NSSI).	A descriptive qualitative design was used. Conventional content analysis was used for data analysis.	18 children, 11 girls and 7 boys, aged 12–18 with depression and NSSI

## Results

### Study characteristics

A total of 40 studies were included. They originated from the United Kingdom (*n* = 11), the USA (*n* = 9), Canada (*n* = 4), China (*n* = 3), Brazil (*n* = 2), Germany (*n* = 2), Australia (*n* = 1), Denmark (*n* = 1), India (*n* = 1), Jordania (*n* = 1), Latvia (*n* = 1), Lithuania (*n* = 1), Norway (*n* = 1), Portugal (*n* = 1) and Thailand (*n* = 1). The years of publication ranged from 2002 to 2024, with the majority published from 2020 onwards (*n* = 22). All studies had obtained ethical approval or addressed ethical considerations through alternative means, with particular emphasis on the involvement of children as participants. The children (*n* = 826) of included studies were 8–19 years old, where most of the studies (*n* = 28) reported participants from thirteen years of age and older. Most studies included both girls and boys (*n* = 33); however, in the majority of the studies (*n* = 34), girls were more represented than boys. For an overview of study characteristics see Table II.

### Key findings related to children’s experiences of their mental ill health

**Figure 2.**
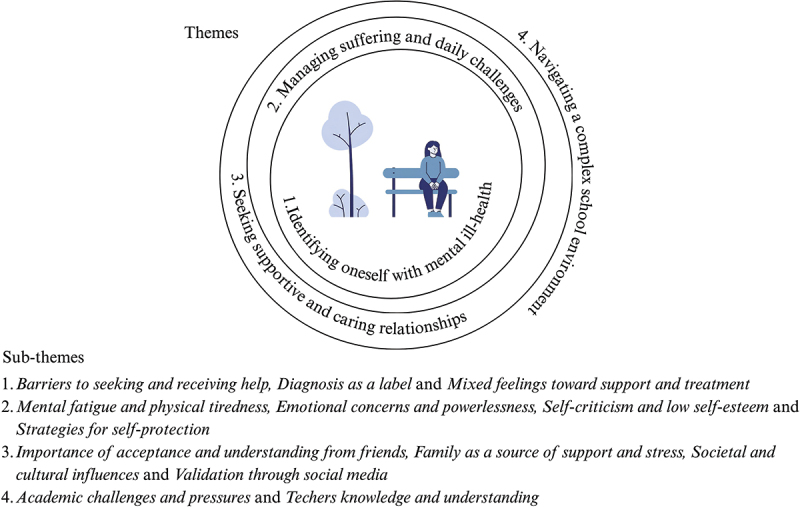
Overview of themes and subthemes.

The experiences of children living with mental ill-health is described in four themes with related sub-themes, see Figure 2.

#### Identifying oneself with mental ill-health

To identify mental ill-health as part of oneself was a journey of mixed emotions. Children experienced fear of being viewed by others as not normal. Receiving a diagnosis, and receiving support and treatment led to conflicting feelings of both helpfulness and hindrances.

##### Barriers to seeking and receiving help

Stigma surrounding mental ill-health created barriers to healthcare, with children reporting that “normal people don’t go to hospital” (Wang et al., 2023). If having to stay at hospital, they feared loss of freedom or negative influence from patients that could be in a worse condition (DeFosset et al., 2017; Gampetro et al., 2012; Wang et al., 2023). Parental attitudes towards healthcare were also influential where some children avoided treatment out of fear their parents would feel embarrassed (Reangsing et al., 2024). Parents’ slowness to recognize mental ill-health, hesitancy to seek help, and dismissal or rejection of their children’s experiences were reported as barriers to healthcare engagement (Platell et al., 2023). Concerns about medication, including fears of being labelled “different” or appearing “crazy” discouraged some children from seeking help or concealing their treatment, even if they believed in its benefits (Dardas et al., 2019; Kranke et al., 2011; Wu et al., 2023).

Practical barriers included financial worries and a lack of information on where to find help. Limited access, particularly to school nurses was also seen as a barrier to receiving help (Corser et al., 2023; Dardas et al., 2019).

##### Diagnosis as a label

Children sometimes struggled to determine whether they were experiencing mental ill-health and relied on healthcare professionals to guide them (Latakienė & Skruibis, 2015). Receiving a diagnosis could feel like a valued part of their identity, helping them to understand themselves and their unique perspectives of the world. However, it could also raise fears of being defined by this “label”, which could redefine their life patterns and self-perception. Some underscored the importance of viewing themselves as individuals *with* a diagnosis, rather than being defined *by* it (Arbour et al., 2023; Viduani et al., 2022). Feeling “abnormal” or “different” led to heightened sensitivity, vulnerability and frustration (Arbour et al., 2023; Bird et al., 2022; Dardas et al., 2019). The fear of judgement was widespread, as children worried about being seen as strange or foolish and sensed others staring or judging them (Halldorsson et al., 2023).

Diagnosis disclosure was another issue. Some children kept their diagnosis a secret out of fear of judgement, social exclusion, or perceived limitations while others reported discomfort with hiding this part of themselves (Arbour et al., 2023; Klauber et al., 2024; Moses, 2010; Shalanski & Ewashen, 2019). Feelings of stigmatization increased when peers labelled their diagnosis as “crazy” (Kranke et al., 2011). Some diagnoses felt easier to disclose compared to others, and sharing brought a mix of relief and anxiety. Moreover, communication about the connection between physical symptoms and mental ill-healthwas unclear, leading to overlooked symptoms and challenges in explaining issues to professionals who specialized in either physical or mental health (Corser et al., 2023).

##### Mixed feelings toward support and treatment

Receiving support and treatment for mental ill-health was linked to various and sometimes conflicting feelings. Cooperation between healthcare and school was welcomed and those with access to school-based health clinics appreciated the support (Gampetro et al., 2012). Therapy and support were valued as they gave the child space to connect with others who had similar challenges and problems (Farmer, 2002). Some children felt more comfortable talking to strangers than family members who might judge them (Platell et al., 2023). However, therapy and support were also associated with negative emotions. Some found it dangerous to open up for others (Li et al., 2022), feeling it would take years to build and become a person with strength enough to build relationship (Shalanski & Ewashen, 2019). For some children, treatment was suggested only after they had been in trouble, and they felt support fell short of their needs (DeFosset et al., 2017; Elsina & Martinsone, 2020). Encounters with healthcare professionals sometimes left children feeling small and insignificant (Midgley et al., 2016). Anonymous online treatment options were appealing, though children worried parents might monitor their browsing history (Platell et al., 2023). Independence in decision-making was desired, but with support from parents and health professionals (Farmer, 2002).

#### Managing suffering and daily challenges

A variety of physical and psychological symptoms affected the children’s well-being. Living with exhaustion and physical issues, and feelings of being emotionally disconnected, powerless and afraid were challenging and affected their self-esteem. However, children also described strategies for self-protection to navigate these challenges.

##### Mental fatigue and physical tiredness

Physical symptoms, perceived as uncontrollable and closely tied to their mental distress, included headaches, dizziness, pain, hypersensitivity, numbness, discoloration, and dislocation (Corser et al., 2023; Farmer, 2002; Weitkamp et al., 2016). The frustration of enduring constant suffering without clear explanations led to a desire for answers. Further, constant exhaustion and an inability to recharge, created a cycle of physical weakness and mental fatigue, despite a full night’s sleep (Higson-Sweeney et al., 2024). Sleep disturbances were linked to accumulated feelings that remained unprocessed or suppressed during the day (Weitkamp et al., 2016), which led to fears and nightmares (Amrtavarshini et al., 2024; Corser et al., 2023). Mental fatigue and physical symptoms, such as extreme tiredness, sometimes became barriers to their potential by, “taking the wind out of their sails” and preventing engagements in activities central to their identity (Corser et al., 2023). This left them unsure of how to be themselves (Farmer, 2002; Higson-Sweeney et al., 2024; Latakienė & Skruibis, 2015). Emotional exhaustion was frequently linked to the suppression of emotions (DeFosset et al., 2017; Farmer, 2002).

##### Emotional concerns and powerlessness

Disconnection from reality was described as living on “autopilot” or observing life rather than actively engaging in it (Tang et al., 2023; Weitkamp et al., 2016). Feelings of emotional disconnection, including numbness, emptiness and an inability to experience joy, diminished children’s enthusiasm for life. These struggles intensified their efforts to maintain or reclaim the identities they felt separated from (Bird et al., 2022; R et al., 2024; Watson et al., 2020; Zhu et al., 2024). Previously enjoyable activities could lose their appeal, contributing to a sense of disinterest. The feeling of being detached from their past selves, struggling to understand their experiences and lacking knowledge of the causes of their mental ill-health, made it hard to explain or control symptoms (Corser et al., 2023; Dardas et al., 2019; Farmer, 2002; Halldorsson et al., 2023; Higson-Sweeney et al., 2024; Latakienė & Skruibis, 2015; Reangsing et al., 2024).

Some children struggled to see beyond their emotional state, feeling lost in chaos and the lack of connection between their mind and soul (Watson et al., 2020; Weitkamp et al., 2016). Feelings of powerlessness made children’s mental ill-health condition worse (Bird et al., 2022) and these feelings could be intensified by constant rumination and a sense of entrapment with an inability to stop worrying (Stänicke, 2021). Or the feeling that something was stuck in their chest that they were unable to get rid of (Dardas et al., 2019). This led to doubts about the possibility of finding relief or imagining a hopeful future (Oliver et al., 2015; Tang et al., 2023). Worries that depression might suddenly return caused constant fear (Woodgate, 2006).

##### Self-criticism and low self-esteem

Mental ill-health linked to lack of motivation and problems with concentration and academic engagement decreased children’s self-esteem (Farmer, 2002; Higson-Sweeney et al., 2024; R et al., 2024; Watson et al., 2020). Self-disappointment and feelings of letting others down were experienced if they did not reach certain goals (Higson-Sweeney et al., 2024; Midgley et al., 2016). Self-hate was also expressed (É. Simões et al., 2021; Weitkamp et al., 2016) as well as feelings of being unworthy of new experiences, improvement or kindness, with descriptions of themselves as “the worst thing ever” (Farmer, 2002; Shalanski & Ewashen, 2019; Shaw et al., 2009) and “a nobody” (R. M. P. Simões et al., 2020). Self-criticism intensified their mental ill-health (Woodgate, 2006), contributing to overwhelming emotional burdens with frequent crying or uncontrolled anger (DeFosset et al., 2017; Farmer, 2002)

##### Strategies for self-protection

Despite various challenges, children could develop strategies to protect themselves and cope with their mental ill-health. Identifying coping needs and finding inner strength were seen as important in building resilience and working with tasks and goals (Arbour et al., 2023; Gampetro et al., 2012). Making sense of mental ill-health challenges could also empower the child and was linked to growing up (Oliver et al., 2015). Taking a pause was helpful when ruminations about life and humanity went too far.

Physical activities, like hillwalking or engaging in intense sports, provided feelings of inclusion and comfort (Farmer, 2002; Reangsing et al., 2024; Watson et al., 2020) and sometimes served as a means to prove to others that they could succeed against all odds (Amrtavarshini et al., 2024). Calming activities like reading were also seen as beneficial (Weitkamp et al., 2016).

Although a need to talk about feelings was expressed, children did not want to be a burden for others (Watson et al., 2020). They sometimes stayed home from school as a way to manage symptoms and emotions (DeFosset et al., 2017). They distanced themselves from friends (Amrtavarshini et al., 2024) and withdrew from social gatherings to avoid social threats (Bird et al., 2022). Children could behave in certain ways to fit in and adapt to their environment (Arbour et al., 2023; Corser et al., 2023; Gampetro et al., 2012; McQueen & Henwood, 2002; Wu et al., 2023), and efforts could also be made to be liked and maintain a good reputation, though they sometimes felt that actions and decisions they made during periods of intense mental ill-health harmed their social image (Halldorsson et al., 2023; Reangsing et al., 2024). Strategies for self-protection also included avoiding eye contact, staying indoors, and observing other’s actions, though this often led to hypervigilance and over-analysis of social cues (Bird et al., 2022; Midgley et al., 2016).

When escape was not possible, blocking out or just enduring negative emotions and experiences was reported as a protective strategy (McQueen & Henwood, 2002; Watson et al., 2020). Children’s outbursts of anger at home, rather than in school, could function as a way to release restrained distress, with minor obstacles sometimes triggering these emotions in a safe family environment (Corser et al., 2023).

Self-injury was also reported as a protective barrier by providing temporary relief from mental pain. This served as a distraction and fostered a sense of control, although it was linked to feelings of shame (Dardas et al., 2019; Stänicke, 2021; Wang et al., 2023; Zhu et al., 2024). Similarly, the ineffectiveness of other coping strategies were described as a factor that could lead children to consider suicide - either as a way to end the need to mask their true emotions (Latakienė & Skruibis, 2015; Zhu et al., 2024) or to stop forcing a smile for others, when they actually wanted to cry (É. Simões et al., 2021).

#### Seeking supportive and caring relationships

Supportive and caring relationships were important factors for the children´s well-being. Seeking acceptance, understanding and support from friends, family and community were central experiences. Validation sought through social media was significant, but also challenging.

##### Importance of acceptance and understanding from friends

Understanding friends, considered “true friends”, helped normalize mental ill-health struggles and provided acceptance (Elsina & Martinsone, 2020; Moses, 2010). Sharing similar experiences about mental ill-health fostered trust and offered comfort, as children often turned to friends first for help, finding acceptance, understanding and relief through conversations about their struggles (Bird et al., 2022; Dardas et al., 2019; Elsina & Martinsone, 2020; Klauber et al., 2024). Being with friends was a valuable escape from inner distress and could enable children to feel that “all is well” and momentarily forget their problems (Farmer, 2002; Weitkamp et al., 2016).

Feelings associated with friends were not always positive. However, Maintaining close friendships were related to feelings of frustration—fearing loss and questioning others´ sincerity due to concerns about their own likeability (Bird et al., 2022; Elsina & Martinsone, 2020). Past betrayals sometimes made them hesitant to trust new friends (Klauber et al., 2024). Friends were described as unnecessary when children were feeling worse or “on the way down” to a depression. They pushed friends away, only to regret it later (Mulfinger et al., 2019; Reangsing et al., 2024; Weitkamp et al., 2016). Conflicts with school friends increased mental ill-health where feelings of being “frozen” or anxious in front of school peers were described (Halldorsson et al., 2023; Latakienė & Skruibis, 2015).

##### Family as a source of support and stress

Confiding in parents calmed fears and supported personal growth, particularly when parents were informed and involved with children´s mental ill-health challenges (Arbour et al., 2023; Dardas et al., 2019; Oliver et al., 2015). Understanding parents sometimes made children feel as if they did not have any problems (Moses, 2010). Yet, despite reassurances from parents, children struggled to see themselves as “good” or “nice” (Wu et al., 2023). Children refrained from sharing their struggles with parents to avoid causing them feelings of worry or guilt (Dardas et al., 2019; Klauber et al., 2024; Platell et al., 2023; Shaw et al., 2009). Parental responses to disclosures of mental ill-health varied: some dismissed them as signs of weakness while others failed to listen or understand or made unhelpful comparisons with siblings. These parental reactions sometimes led to emotional distance, causing children to feel uncared for, less loved or overly protected (Arbour et al., 2023; Gampetro et al., 2012; Moses, 2010; Viduani et al., 2022).

Extended family could also be a source of stress. Some children felt isolated, unloved or mocked within their larger family circles, which brought feelings of not fitting in (Farmer, 2002; Shaw et al., 2009).

##### Societal and cultural influences

Societal and cultural attitudes shaped children’s experiences of mental ill-health. Physical illnesses were more accepted and understood than mental ill-health, which children sensed lacked recognition as “real illness” (Higson-Sweeney et al., 2024; Mulfinger et al., 2019). Children had experienced peers gossiping that mental ill-health was due to evil acts, either personal or ancestral (Wang et al., 2023). Dismissal as attention seekers was also described (Singleton et al., 2016). Boys felt that they lost their masculinity, by feeling “soft” and were pressured to “toughen up” (McQueen & Henwood, 2002). Girls of colour felt they were expected to feel secure in their bodies despite if they were overweight, making eating disorders feel even more isolating. Girls from impoverished families feared being labelled bewitched if they revealed their mental ill-health (Kranke et al., 2012). Children could attend school, even though depressed, to avoid household responsibilities (Dardas et al., 2019). Anxiety about perceived looks of curiosity or judgement from others reinforced their sense of not fitting in (Bird et al., 2022; Mulfinger et al., 2019). Societal and cultural beliefs sometimes shaped how children viewed treatment options, with some seeing medication as a “gift from God”, while others preferred to manage independently (Kranke et al., 2012).

##### Validation through social media

Positive connections and validation were sought online (Kline et al., 2023; Singleton et al., 2016). Social media was both a source of connection to others and a potential challenge. It served as a distraction from the world during periods of isolation, and children found comfort in knowing they were not alone (Kline et al., 2023). Online friends were a helpful diversion and offered reassurance that others are “in the same boat”; restricted access to cell phones could therefore cause anxiety and negative emotions (Singleton et al., 2016). Others described a comparison and competition of mental ill-health on social media. Tweets on social media drew attention from school friends but sometimes caused insecurity or bullying (Jha et al., 2024; Klauber et al., 2024). Disturbing videos with inappropriate content such as ways to commit suicide were introduced to some social media users.

##### Navigating a complex school environment

Academic challenges were seen as pressures, but challenges and routines were also necessary to feel adequate and useful. The relationship with school and teachers could be both a source of comfort or stress.

##### Academic challenges and pressure

School itself was experienced as a complex environment. While it served as a safe haven and motivator for some, it was exhausting and draining for others (R et al., 2024). Mental ill-health was linked to school requirements, particularly for those children who felt pressured to excel academically. This led to resentment towards the need to be “best in class” (Stänicke, 2021). Moreover, academic overload often hindered building relationships (Dardas et al., 2019; Elsina & Martinsone, 2020; Weitkamp et al., 2016; Wisdom & Green, 2004; Wu et al., 2023; Zhu et al., 2024). Poor academic performance caused some to ruminate on feelings of inadequacy (Latakienė & Skruibis, 2015; Oliver et al., 2015), and changes in school routines or schools often intensified mental health struggles (É. Simões et al., 2021). Various concerns children referred to when discussing school and future development were heightened awareness of the risks that come with growing older (Bird et al., 2022), fear of failure (Halldorsson et al., 2023), anxiety about future careers (Gampetro et al., 2012) and general uncertainty about their ability to handle future challenges (Reangsing et al., 2024).

##### Teachers knowledge and understanding

Children wished for teachers to understand mental ill-health in order to be better equipped to deal with their students’ crises (Corser et al., 2023; Klauber et al., 2024). Being ignored or overly monitored by teachers, treated differently or given leniency compared to peers without mental ill-health led to feelings of awkwardness (Moses, 2010; Mulfinger et al., 2019). Teachers were sometimes perceived as joking about mental ill-health (Vallani et al., 2023), overlooking the root causes of students’ struggles or relying on discipline rather than identifying mental health needs (Dardas et al., 2019; Elsina & Martinsone, 2020). Children were left with feelings of being misunderstood and unsupported when teachers were too focused on teaching only (Dardas et al., 2019; DeFosset et al., 2017).

## Discussion

Results revealed that children’s experiences of mental ill-health were marked by an everyday struggle of living with mixed emotions, where diagnoses and treatment brought both support and challenges. They faced physical and emotional difficulties such as pain and exhaustion while striving to develop strategies for self-protection. Supportive relationships with family, friends, teachers and community were crucial. Social media and school served as both sources of strength and stress.

The results indicate that children with mental ill-health face challenges in understanding and articulating their experiences and they have a need for both understanding from others and social support. Children face numerous life challenges, such as academic failures, relationship problems, and negative feelings about themselves and their performance; these significantly impact their well-being and mental health, often making life feel overwhelming and leading to stress reactions (Hellström & Beckman, 2021). There appears to be a fine line between what can be considered typical adolescent experiences and the onset of mental ill-health. Like children in general (Beckman et al., 2023), those experiencing mental ill-health struggle to grasp the nature of their mental health and distinguish mental health problems from everyday health concerns and challenges. Additionally, experiences of health and health problems may coexist, reflecting the holistic nature of the “lived body”, as described by the philosopher Merleau-Ponty (2002). Physical symptoms, however, may serve as a way for children to express their mental ill-health. Fatigue, physical exhaustion and pain might all be signs of psychosomatic status and for some children these symptoms might be easier to talk about and to seek help with. Stress from school and home environments is a common underlying factor for physical and psychosomatic symptoms (Public Health Agency of Sweden, 2018), highlighting the need for greater understanding and support from adults. Health care professionals should therefore evaluate physical issues and pain to a greater extent when meeting children with mental ill-health. Midgley et al. (2017) note that children seek to understand their ill-health as a means to restore their sense of self and manage their emotions and challenges. To be understood, to receive help and to be involved in decision making on how to handle mental ill-health may support recovery for these children. To enable recovery: health care should strive for quick and available contact for those showing symptoms or seeking help, to make sure that delayed or unavailable health care contacts do not act as barriers for children to seek help (Darcy, Råberus et al., 2024).

This review recounts various strategies children use to cope with their mental ill-health such as turning to nature or calming activities. These strategies empower them in managing their difficulties and enhancing their well-being. Hellström and Beckman (2021) highlight adolescents’ needs for coping strategies and means of managing negative stereotypes and self-blame. Listening to children’s individual voices is crucial in paediatric nursing care, as it helps identify their unique needs and strengths (Darcy, Israelsson-Skogsberg et al., 2024; Kleye et al., 2021). This should also be true in caring for children living with mental ill-health. Including the voices of children with mental ill-health in care and research is essential for providing effective support and enabling children to navigate difficult situations. Children’s experiences of health and ill-health cannot be fully understood in isolation from their developmental stage and broader life context (Darcy, Israelsson-Skogsberg et al., 2024). Children’s perceptions and coping mechanisms may be shaped by their living phase, which influences how they interpret and express mental health challenges.

Results of this review show that mental ill-health was linked to emotional concerns with feelings of being disconnected and powerless in their environments. Children struggled with expectations they and others placed on themselves, leading to self-hate and lowered self-esteem. Stressors and demands can leave adolescents feeling overwhelmed, with little or no opportunity for recovery (Tang et al., 2023). The inability to plan or achieve goals can further intensify feelings of mental ill-health. Poon and Loh (2024) found that adolescents often attempt to manage their mental ill-health by themselves, which can make thoughts of self-harm and suicide challenging to navigate. In general, adolescence is a pivotal stage of development marked by numerous challenges, such as academic pressure, navigating relationships with parents and peers, and forming one’s identity (Tetzner et al., 2017). Social media was reported by children to function as an extension of traditional support systems where they searched for the validation with others. Children’s anxiety increased when adults removed the connection to social media. Moreover, some children mentioned a desire for receiving internet-based mental health care to be able to search for help regardless of their parents’ attitudes. Societal and political discussions often lift problems with social media and proclaim screening time restrictions. However, social media may offer children a more accessible and less intimidating way to explore both mental health resources and connect with others (Naslund et al., 2020). In line with the current findings, it has been stressed that social media usage has both positive and negative influences (Weinstein, 2018). Children of current review expressed that they would like to try mental healthcare through internet platforms, but were afraid that their parents would browse their history. However, recent research reveals that parental guidance of online behaviours can be protective (Mabaso et al., 2024). Both these statements should be taken into consideration in clinical work when discussing decision-making with children on how they want their mental ill-health to be treated.

The importance of peer relationships was underscored, as children tend to turn to friends first for understanding. This aligns with findings from Roach et al. (2021), who established that peer relationships can reduce anxiety and depression. However, challenges include the fact that children may lack the necessary skills to support their friends effectively, and supportive peers may face an emotional burden. These challenges highlight the need for interventions that promote mental health literacy among children to enable effective peer support without overburdening the helper.

The findings of the current scoping review also reveal that the perception of the teacher’s role warrants further discussion. The notion that a teacher serves solely as a pedagogical support, rather than a source of emotional or mental support, may vary depending on the individual teacher and pupil. Addressing mental health and ill-health as a subject in schools is a discussion that gains attention in political discussions as the implementation of school-based mental health promotions into school-curricula requires investments and actions from governments and schools (Margaretha et al., 2023). Teachers need both knowledge of mental ill-health and awareness of individual children’s pressures and needs. When mental health promotion is incorporated into the school-curricula it can lead to increased awareness and knowledge, reduced stigma, improved help-seeking behaviours, enhanced overall mental health among students and better training for teachers to support students effectively (Milin et al., 2016). Facilitating mental health as an integrated school subject could potentially also reduce challenges with the school environment as reported by children in current scoping review. Furthermore, mental health promotion programmes could foster a sense of collaboration between school and health care services, ultimately working in the child’s best interest to facilitate well-being.

### Strengths and limitations

The preregistration in OSF demonstrated transparency and commitment to methodological rigour, while the application of MMAT (Hong et al., 2018) ensured a systematic quality assessment of included studies. To ensure a comprehensive search across multiple disciplines related to children’s mental health the databases PubMed, CINAHL, and PsychINFO were chosen. The large number of search results identified during the scoping review reflects the use of a broad search strategy, which is in line with the principles of scoping review methodology (Joanna Briggs Institute, 2020). The objective was not to focus on a specific diagnosis, age or sex, but to capture a broad view of children’s mental ill-health experiences. Inclusion criteria concerning publications from the year 2000 and onward allowed us to examine children’s mental health in a context that remains relatively contemporary while allowing for a broad temporal scope. Interestingly, few studies were published in the early 2000s, but there has been a sharp increase in publications in recent years, particularly in 2023. This trend reflects both a growing research focus on the topic and potential delays in publication processes caused by the COVID-19 pandemic. Moreover, the pandemic has heightened global awareness of children’s mental health, likely contributing to the surge in related studies during this period.

There are limitations with the current scoping review to consider. One potential is the exclusion of grey literature as relevant findings from such sources may have been overlooked. It might also have been qualitative results that could have contribute to this review excluded due to the exclusion criteria of mixed-methods. This scoping review mainly captured girl’s experiences, which is in line with available literature showing that increased stress and anxiety is mainly associated with girls’ well-being. Boys and young men’s mental ill-health is a rather unexplored area (Public Health Agency of Sweden, 2018). While the inclusion criterion aimed to capture diverse perspectives, the lack of representation from certain global regions could reflect underdeveloped research infrastructure in some areas, biases in database indexing, or cultural stigmas surrounding mental health. This may limit the generalizability of the results. It was not an equal blend of age and sex, and it would be of significance in further research to perform qualitative studies with boys and with younger children as there is a knowledge gap in understanding well-being and mental ill-health in younger children. Identifying and treating children with early symptoms correctly so that they do not develop more serious mental ill-health should be a focus for services, going forward. The research team suspected there would be limited studies involving younger children and the hypothesis was proved accurate based on the results.

## Conclusions

This study highlights the complex everyday lives of children with mental ill-health, their desire to manage and comprehend their difficulties and need for understanding, tailored strategies and supportive relationships. Although a diagnosis may put a label to their ill-health problems, it is still something that can be hard to navigate and talk about. Children with mental ill-health need to understand their feelings and need support and strategies to manage their mental ill-health and feel well. They sometimes hide their true emotions and express a desire for supported yet independent decision-making. Friends, parents, teachers and health care professionals can provide understanding and support for these children. Striving for wellbeing requires open and trustful relationships free from judgemental or critical attitudes. By focusing on children’s lived experiences, this scoping review contributes to the literature by emphasizing the central role of children’s voices in matters of mental health and ill-health.

Further research is needed, especially qualitative research exploring the experiences of younger children and healthcare professionals’ perspectives on effective approaches for talking with and supporting children with mental ill-health. Future research should also explore how developmental aspects interact with experiences of mental ill-health and healthcare professionals, as this could provide deeper insights into specific needs and support mechanisms for children living with mental ill-health.

Health care professionals should prioritize assessing physical symptoms and pain in children with mental ill-health and ensure accessible support with enough time. Integrating mental health education into schools and strengthening collaboration between schools and health care services could enhance well-being and mental health literacy. Additionally, discussions on treatment preferences should consider the influence of social media on children’s decision-making. Incorporating children’s voices in care and research is essential for effective interventions.

## Supplementary Material

Supplementary_file_1_PRISMA_ScR_Checklist_Childrens_experiences_of_living_with_their_mental_illhealth_a_scoping_review.docx

Supplementary_file_2_query_string_Childrens_experiences_of_living_with_their_mental_illhealth_a scoping review.docx

Legends_for_Figures_Tables_Supplements.docx
