# Supplementary material for: Children’s experiences of living with their mental ill-health - a scoping review
Source: Int J Qual Stud Health Well-being. 2025 May 7;20(1):2501682. doi: 10.1080/17482631.2025.2501682 (PMC12064100; doi:10.1080/17482631.2025.2501682)
Supplement: Supplementary_file_2_query_string_Childrens_experiences_of_living_with_their_mental_illhealth_a scoping review.docx [file ZQHW_A_2501682_SM9751.docx]

PUBMED

("Qualitative Research"[Mesh] OR "qualitative interview*"[Title/Abstract] OR "qualitative study"[Title/Abstract]) AND (attitude*[Mesh] OR attitude*[Title/Abstract] OR perspective*[Title/Abstract] OR experience*[Title/Abstract]) AND ("Child"[Mesh:NoExp] OR "Adolescent"[Mesh] OR child[Title/Abstract] OR children*[Title/Abstract] OR adolescen*[Title/Abstract]) AND ("Mental Disorders"[Mesh] OR "Mental Health"[Mesh] OR "Mental Disorders"[Title/Abstract] OR "mental illness"[Title/Abstract] OR "mental ill health"[Title/Abstract] OR "mental health"[Title/Abstract])

CINAHL

((MH "Qualitative Studies+") OR TI “qualitative interview*” OR AB “qualitative interview*” OR TI “qualitative study” OR AB “qualitative study”)

**AND**

(MW attitude* OR TI attitude OR AB attitude OR TI experience OR AB experience OR TI perspective OR AB perspective)

**AND**

((MH "Mental Disorders+") OR (MH "Mental Health") OR TI "mental disorder*" OR AB "mental disorder*" OR TI "mental health" OR AB "mental health" OR TI "mental illness" OR AB "mental illness" OR TI "mental ill health" OR AB "mental ill health")

**AND**

((MH "Child") OR (MH "Adolescence") OR TI child OR AB child OR TI children OR AB children OR TI adolescen* OR AB adolescen*)

PsycInfo

(MAINSUBJECT.EXACT.EXPLODE("Qualitative Methods") OR tiab("qualitative interview*") OR tiab("qualitative study")) AND ((MAINSUBJECT.EXACT.EXPLODE("Experiences (Events)") OR MAINSUBJECT.EXACT.EXPLODE("Attitudes")) OR tiab(attitude*) OR tiab(experience*) OR tiab(perception*)) AND (tiab(child OR children* OR adolescen*) OR age.exact("School Age (6-12 Yrs)" OR "Adolescence (13-17 Yrs)" OR "Childhood (birth-12 Yrs)")) AND ((MAINSUBJECT.EXACT.EXPLODE("Mental Health") OR MAINSUBJECT.EXACT.EXPLODE("Mental Disorders")) OR tiab("mental disorder*" OR "mental health" OR "mental illness" OR "mental ill health")) AND (stype.exact("Scholarly Journals") AND yr(2000-2029) AND PEER(yes))
