# Supplementary material for: Children’s experiences of living with their mental ill-health - a scoping review
Source: Int J Qual Stud Health Well-being. 2025 May 7;20(1):2501682. doi: 10.1080/17482631.2025.2501682 (PMC12064100; doi:10.1080/17482631.2025.2501682)
Supplement: Legends_for_Figures_Tables_Supplements.docx [file ZQHW_A_2501682_SM9750.docx]

**Legends for Figures, tables and supplements:**

**Supplementary file 1**: PRISMA ScR Checklist, *Children’s experiences of living with their mental ill-health – a scoping review.*

**Table 1.** Population, Concept and Context (PCC)

**Supplementary file 2**: The query-strings for each of the databases used; Cinahl, PubMed and PsychInfo. Date the most recent search was executed: 240917. *Children’s experiences of living with their mental ill-health – a scoping review.*

**Figure 1.** Flow chart of the literature search and screening according to PRISMA (Tricco et al. 2018)

**Table 2.** Characteristics and summary of included studies

**Figure 2.** Overview of themes and subthemes
